# Future Directions in Maintenance Therapy in Multiple Myeloma

**DOI:** 10.3390/jcm10112261

**Published:** 2021-05-24

**Authors:** Sarah A. Holstein, Vera J. Suman, Jens Hillengass, Philip L. McCarthy

**Affiliations:** 1 Department of Internal Medicine, Division of Oncology and Hematology, University of Nebraska Medical Center, Omaha, NE 68198, USA; 2 Department of Health Sciences, Division of Quantitative Health Sciences, Mayo Clinic, Rochester, MN 55905, USA; suman@mayo.edu; 3 Myeloma Service, Roswell Park Comprehensive Cancer Center, Department of Medicine, Buffalo, NY 14203, USA; jens.hillengass@roswellpark.org; 4 Roswell Park Comprehensive Cancer Center, Transplant and Cellular Therapy Program, Department of Medicine, Buffalo, NY 14203, USA; philip.mccarthy@roswellpark.org

**Keywords:** maintenance, lenalidomide, multiple myeloma, minimal residual disease, overall survival, transplant

## Abstract

Autologous stem cell transplantation (ASCT) has been a backbone of therapy for newly diagnosed patients with multiple myeloma eligible for high-dose therapy for decades. Survival outcomes have continued to improve over time, in part because of the incorporation of highly effective induction regimens prior to ASCT as well as post-ASCT maintenance therapy. Randomized phase III clinical trials have helped establish lenalidomide maintenance as a standard of care. However, as nearly all patients will eventually experience disease relapse, there continues to be significant interest in developing novel maintenance strategies to improve upon lenalidomide maintenance. In this review, we summarize the available evidence for the use of immunomodulatory drugs, proteasome inhibitors, and monoclonal antibodies as post-ASCT maintenance therapies as well as discuss future directions and unanswered questions in the field.

## 1. Introduction

The outcomes of patients with multiple myeloma (MM) who are eligible for high-dose chemotherapy and autologous stem cell transplant (ASCT) are continuing to improve because of the advancements in pre-ASCT induction regimens with the introduction of immunomodulatory drugs (IMIDs) and proteasome inhibitors (PIs) and in post-ASCT maintenance strategies with the incorporation of lenalidomide. A major aim of maintenance therapy is to significantly delay disease recurrence that would require re-treatment and preferably to prolong overall survival (OS) without incurring excessive toxicity. In this review, we present an overview of the data that have led to current standards of care as well as focus on the ongoing studies that will likely shape the field over the next decade. Key unanswered questions in the field include the optimal duration of maintenance therapy, whether one drug is sufficient or whether multi-agent therapy should be used, whether response-adapted approaches can guide maintenance therapy, as well as whether cytogenetic risk or other disease-based characteristics can guide maintenance therapy choices.

## 2. Single-Agent Maintenance Strategies

### 2.1. Immunomodulatory Drugs

The IMiD class of drugs, which includes thalidomide, lenalidomide, and pomalidomide, are oral medications with once-daily dosing. The activity of these agents in the relapsed/refractory setting as well as in the newly diagnosed setting (thalidomide and lenalidomide) has led to multiple studies evaluating their use as a maintenance therapy in the post-ASCT setting. As discussed below, single-agent lenalidomide is now considered a standard of care based on multiple phase III studies and is currently serving as the control arm for multiple maintenance studies evaluating lenalidomide-based combinations.

#### 2.1.1. Thalidomide

Several studies evaluated the impact of post-ASCT thalidomide maintenance. These studies differed with respect to initial dose used (range, 50–400 mg) and whether thalidomide was used in combination with glucocorticoids. Some of the studies did demonstrate improved event-free survival (EFS) or OS, whereas others did not (reviewed by McCarthy and Hahn [1]). A meta-analysis of five of these studies demonstrated an OS benefit with thalidomide maintenance (odds ratio, 0.75; 95% CI, 0.64–0.87; *p* < 0.001) [2]. However, the median duration of treatment in several studies was less than 18 months, treatment-related toxicities (including neuropathy, thromboembolic events, sedation, and constipation) leading to discontinuation in 6–46% [2,3,4,5]. With the advent of lenalidomide, thalidomide is now rarely used in the post-ASCT setting in the United States. It is still utilized in countries with less access to lenalidomide for maintenance.

#### 2.1.2. Lenalidomide

Four randomized phase III studies have evaluated lenalidomide maintenance vs. observation/placebo post-ASCT [6,7,8,9,10]. The details of these studies are shown in Table 1. While there were differences in patient populations (especially different induction regimens), study design (the IFM 2005 study included two cycles of consolidation with full-dose lenalidomide prior to randomization), and dosing schedule (continuous vs. 21/28 days dosing), the four studies had very similar median time to progression (TTP)/progression free survival (PFS) outcomes with hazard ratios (HRs) ranging from 0.47 to 0.57 in favor of the lenalidomide arm [6,7,8,9,10]. A meta-analysis of the first three studies to be conducted (CALGB 100104, IFM 2005, and GIMEMA-RVMM-PI209) revealed a median PFS of 52.8 months for the lenalidomide group compared to 23.5 months for the placebo/observation group (HR, 0.48; 95% CI, 0.41–0.55) [11]. None of the studies were powered to evaluate OS as a primary endpoint, but in the meta-analysis, a significant OS benefit was reported (median not reached vs. 86.0 months; HR, 0.75; 95% CI, 0.63–0.90; *p* = 0.001) for lenalidomide vs. placebo/observation [11]. The addition of prednisone to lenalidomide did not improve outcomes compared to lenalidomide maintenance alone in the EMN-441 study [12].

The primary adverse events associated with lenalidomide maintenance include cytopenias, rash, and diarrhea. A pooled analysis of the CALGB and IFM studies showed that the percentage of patients experiencing at least one treatment-emergent adverse event (TEAE) leading to discontinuation was 29% in the lenalidomide group compared to 12% in the placebo group [11]. As discussed below in greater detail, an increased risk of second primary malignancies (SPMs) has also been noted in association with lenalidomide maintenance post-ASCT. However, cumulative incidence risk analyses have demonstrated that the risk of death from MM (which is significantly decreased with lenalidomide maintenance) remains much higher than the risk of death from an SPM [8,10,11]. Based on these data, both the United States Federal Drug Administration (FDA) and the European Medicines Agency approved lenalidomide as maintenance therapy post-ASCT in 2017. 

A second meta-analysis including the three prior studies along with the maintenance portion of the Myeloma XI study found an overall HR of 0.47 (95% CI, 0.41–0.54) for PFS and 0.72 (95% CI, 0.56–0.91) for OS [10]. Subgroup analysis from the McCarthy et al. meta-analysis revealed benefit with lenalidomide regardless of age, sex, International Staging System stage, response after ASCT, or whether patients received a lenalidomide-containing induction regimen [11]. Less than half of the subjects in the meta-analysis had diagnostic cytogenetic risk information (no information was available from the CALGB 100,104 study), and thus with limited numbers of patients with high-risk cytogenetic features (n = 56 for the lenalidomide group, n = 36 for the placebo/observation), a statistically significant improvement in PFS was not observed (HR, 0.86; 95% CI, 0.53–1.40) [11]. However, the Myeloma XI study did collect cytogenetic information on a greater number of patients (774 out of the 1971 total patients (transplant-eligible and transplant-ineligible)). This study reported benefit for lenalidomide across all three cytogenetic risk groups for the transplant-eligible subgroup: median PFS not reached vs. ~30 months for standard-risk (no high-risk features); ~54 months vs. ~24.5 months for high-risk (one high-risk feature); ~22.5 months vs. ~7.5 months for ultra-high-risk (more than one high-risk feature) [10]. 

#### 2.1.3. Pomalidomide

There are limited data regarding the use of pomalidomide maintenance and these arise primarily from the salvage ASCT setting. A single-institution experience reported a median PFS of 12 months among seven patients treated with pomalidomide maintenance post-ASCT [13]. These patients had a median of four prior lines of therapy. The median PFS was 12 months. The phase II EMN011 study enrolled patients who progressed on the EMN02/HO95 study (and are refractory to lenalidomide and bortezomib) and involves KPD induction followed by salvage ASCT, KPD consolidation, and then a randomization to either pomalidomide maintenance (4 mg days 1–21) or pomalidomide in combination with dexamethasone (40 mg weekly) [14]. Results from the maintenance component of the study await longer follow-up.

### 2.2. Proteasome Inhibitors

PIs have been widely used in both the newly diagnosed and relapsed/refractory settings, thus it is not surprising that there has been interest in the potential for these agents in the post-ASCT maintenance setting. However, with the exception of ixazomib in the TOURMALINE-MM3 study discussed below, these agents have not been studied in placebo-controlled trials.

#### 2.2.1. Bortezomib

Those who have advocated for the use of single-agent bortezomib maintenance post-ASCT have done so primarily on the basis of the HOVON-65/GMMG-HD4 trial. This study evaluated the impact of incorporating bortezomib into induction and maintenance. Newly diagnosed patients were randomized to either VAD induction (vincristine, doxorubicin, and dexamethasone) followed by ASCT (single or tandem) and thalidomide maintenance or PAD induction (bortezomib, doxorubicin, and dexamethasone) followed by ASCT (single or tandem) and bortezomib maintenance [15]. For the maintenance component, bortezomib was administered every other week for up to two years. Given the study design, a direct comparison of the two maintenance strategies was not possible. It was noted, however, that when PFS was calculated from time of ASCT, a statistically significant benefit was observed with bortezomib. Subset analysis showed that bortezomib improved PFS/OS for patients with a creatinine greater than two at presentation and for patients with del(17p) but not for patients with t(4; 14) or gain(1q21) [16]. While this led to some experts recommending bortezomib post-ASCT maintenance for patients with high-risk cytogenetics, it is important to note the comparator arm of thalidomide in this study. In the Myeloma IX trial, no significant improvement in PFS and inferior OS were seen with thalidomide relative to observation in patients with high-risk features [2]. In this context, it is difficult to discern the impact that bortezomib maintenance has in high-risk disease.

#### 2.2.2. Ixazomib

The TOURMALINE-MM3 was a randomized phase III study comparing ixazomib to placebo maintenance therapy post-ASCT [17]. Patients were eligible if they had achieved at least a partial response after completing induction therapy and a MEL200 ASCT within 12 months of diagnosis. Induction therapy must have included a PI or an IMiD. Randomization occurred in a 3:2 manner (ixazomib:placebo) with stratification based on induction regimen (PI without IMiD vs. IMiD without PI vs. PI + IMiD), ISS at diagnosis, and response post-ASCT (CR or VGPR vs. PR). Only 11% of patients received an induction regimen that did not contain a PI, with 30% receiving PI + IMiD induction. Eighteen percent of patients had high-risk cytogenetic abnormalities (defined as del(17p), t(4; 14), and t(14; 16)). Treatment consisted of 3 mg of ixazomib on days 1, 8, and 15 of a 28-day cycle with a dose escalation to 4 mg permitted after cycle 4. Maintenance therapy was to be continued for up to 24 months (26 cycles). With a median follow-up of 31 months, the median PFS (from time of randomization) was 26.5 months (95% CI, 23.7–33.8) in the ixazomib group vs. 21.3 months (95% CI, 18.0–24.7) in the placebo group (HR, 0.72; 95% CI, 0.58–0.89; *p* = 0.0023). A trend towards benefit was observed in the high-risk cytogenetic subgroup (HR, 0.62; 95%, CI 0.38–1.02)). Amongst those patients who were minimal residual disease (MRD)-positive (10^−5^ by flow) post-ASCT, 12% in the ixazomib group and 7% in the placebo group converted to MRD negativity during maintenance therapy. Of those who were MRD-negative post-ASCT, 62% in the ixazomib group maintained MRD negativity compared to 51% in the placebo group. After 33/55 (60%) patients in the ixazomib group developed herpes zoster compared to 12/47 (26%) in the placebo group, a protocol amendment requiring prophylaxis was instituted. Thereafter, the number of patients diagnosed with herpes zoster decreased dramatically (6/339 (2%) in the ixazomib group and 2/212 (1%) in the placebo group). The most common AEs included neutropenia, thrombocytopenia, anemia, infection, nausea, diarrhea, vomiting, and rash. Three percent of patients in each arm (n = 12 for ixazomib; n = 8 for placebo) were diagnosed with an SPM. While the TOURMALINE-MM3 had statistically significant results and met its primary endpoint, it can be argued that the improvement of median PFS by only 5.2 months is not clinically significant, particularly when compared to the ~29-month improvement achieved with lenalidomide maintenance [11]. In this context, we would recommend considering ixazomib maintenance only if there was a strong contra-indication to lenalidomide (e.g., serious rash, evidence of emerging myelodysplastic syndrome).

#### 2.2.3. Carfilzomib

To date, a phase III randomized trial evaluating single-agent carfilzomib maintenance therapy post-upfront ASCT has yet to be conducted. However, several studies have evaluated carfilzomib maintenance following salvage ASCT. Costa et al. reported the results of a phase I/II trial evaluating the addition of carfilzomib to high-dose melphalan with ASCT and then 12 cycles of carfilzomib maintenance in a relapsed/refractory patient population [18]. Two different schedules of carfilzomib maintenance were evaluated: 36 mg/m^2^ days 1, 8, and 15 (A) or days 1, 2, 15, and 16 (B). Interestingly, patients received two cycles of each schedule and then were given a choice of which one to use for the remaining cycles. Of the 27 patients who started maintenance, the 12-month PFS rate was 66.7%. Equal numbers of patients chose to complete maintenance with schedule A vs. B. The most common AEs (any grade) during maintenance included infection (26%), nausea/vomiting (22%), and fatigue (19%), with the most common grade 3/4 AEs being infection (15%), acute kidney injury (11%), and neutropenia (11%) [18]. 

The CARFI trial conducted by the Nordic Myeloma Study Group was a randomized phase II study in which patients in first relapse following upfront ASCT received salvage therapy with carfilzomib/cyclophosphamide/dexamethasone followed by ASCT [19]. Two months post-ASCT the patients were randomized to either observation (n = 86) or to maintenance with carfilzomib (27/56 mg/m^2^ every other week) in combination with dexamethasone (Kd) (n = 82). The primary endpoint of the study was difference in TTP after the upfront ASCT vs. the salvage ASCT, however a second primary endpoint involved a comparison of the TTP of the two maintenance strategies. Notably, none of the patients had received maintenance therapy after their first ASCT. The median TTP from randomization was 28.8 months (95% CI, 24.4–not reached) in the Kd arm vs. 18.5 months (95% CI, 14.3–22.0) in the observation arm (HR, 0.42; 95% CI, 0.26–0.68; *p* = 0.0003) [19]. There were 53 SAEs reported in 25 patients in the Kd maintenance group compared to 33 SAEs in 21 patients in the observation group, with the majority of AEs being infections in both groups [19].

### 2.3. Daratumumab

Only one phase III study has evaluated single-agent daratumumab in comparison to observation. The CASSIOPEIA study primarily evaluated the addition of daratumumab to the bortezomib/thalidomide/dexamethasone (VTD) backbone at induction and consolidation post-ASCT [20]. However, there was a second randomization postconsolidation to single-agent daratumumab maintenance (every 8-week dosing up to 2 years) vs. observation. Results from a preplanned interim analysis reported in a press-release (October 2020) provided an HR of 0.53 (95% CI, 0.42–0.68; *p* < 0.0001) in favor of daratumumab over observation.

## 3. Combination Therapy Strategies

While multiple randomized phase III studies have demonstrated significant PFS benefit for lenalidomide maintenance relative to observation/placebo, nearly all patients will relapse and require additional therapy. In addition, as noted above, the outcomes for patients with high-risk cytogenetic features remain inferior to those with standard risk features. Thus, there has been significant interest in the potential role for lenalidomide-based combination maintenance therapy. While the goal of such a strategy is to improve time to progression and hopefully ultimately prolong OS, the downsides of combination therapy include the potential for increased burden to the patients, in terms of toxicities, time away from work/family, and finances.

### 3.1. Lenalidomide + Bortezomib

Nooka et al. reported the results of a single-institution experience with lenalidomide/bortezomib/dexamethasone (RVd) maintenance in 45 patients with high-risk disease [21]. Maintenance consisted of lenalidomide (10 mg/day days 1–21) with weekly bortezomib/dexamethasone for up to 3 years followed by single-agent lenalidomide. High-risk disease was defined as del(17p), del(1p), t(4; 14), and t(14; 16) or primary plasma cell leukemia. The median PFS was 32 months, and no patient discontinued maintenance due to adverse events although 40% required dose modifications [21]. In a more recent publication from this group that evaluated the outcomes of 1000 patients who received RVd induction, the median PFS of patients with high-risk disease who received PI/IMiD maintenance therapy was 40.3 months with a median OS of 78.2 months [22].

### 3.2. Lenalidomide + Ixazomib

The combination of lenalidomide and ixazomib has an appeal because of the all-oral nature. However, there are limited prospective data supporting its use. A retrospective, single-institutional study reported that the median PFS had not been reached among nine patients treated with lenalidomide/ixazomib in the maintenance setting (seven in the post-ASCT maintenance setting), with a median follow-up time of 14 months [23]. Patel et al. have reported the preliminary findings from a single-arm phase II study evaluating the combination of lenalidomide/ixazomib as post-ASCT maintenance therapy in newly diagnosed patients. Ixazomib was administered at a dose of 4 mg on days 1, 8, and 15, while lenalidomide was started at 10 mg days 1–21 (with escalation to 15 mg allowed). The protocol was subsequently amended to lower the starting dose of ixazomib to 3 mg. A total of 64 patients were enrolled. With a median follow-up of 37.8 months, the median PFS was not yet reached and the estimated 2-year PFS was 81% [24].

### 3.3. Lenalidomide + Carfilzomib

Recently, the preliminary results from the maintenance portion of the randomized phase II FORTE study were presented [25]. This study included a second randomization that occurred post-consolidation. Patients were randomized to either single-agent lenalidomide (R) maintenance or lenalidomide in combination with carfilzomib (KR). In this study, lenalidomide was continued until progression while the carfilzomib was stopped after 2 years. Carfilzomib was initially dosed at 36 mg/m^2^ on days 1, 2, 15, and 16 of a 28-day cycle but then was changed to 70 mg/m^2^ on days 1 and 15 following a protocol amendment. In both arms, lenalidomide was dosed at 10 mg/day days 1–21 of the 28-day cycle. An improvement in PFS (from second randomization) was seen in the KR arm compared to the R arm with the 30-month PFS rate of 81% (KR) vs. 68% (R) (*p* = 0.0026) [25]. A higher rate of conversion to MRD negativity was observed in the KR arm (46% vs. 32%), but a higher rate of nonhematological AEs was also reported in the KR arm (27% vs. 15%, grade 3 or higher). This study is ongoing, and longer follow-up is needed to determine whether there is particular benefit of KR maintenance for specific subsets of patients.

### 3.4. Lenalidomide + Vorinostat

There has been interest in incorporating histone deacetylase (HDAC) inhibitors into the post-transplant maintenance setting. Sborov et al. reported the results of a single-institution phase I study evaluating different doses of vorinostat (200–400 mg/day administered on days 1–7 and 15–21) in combination with lenalidomide (days 1–21) [26]. This study also included dose escalation of lenalidomide to 25 mg. After 12 months of treatment, the median dose of lenalidomide was 5 mg and vorinostat was 200 mg [26]. In longer-term follow-up, the median PFS was reported to be 64.3 months (21.7–not reached) and median OS was not yet reached [27]. 

Following a protocol amendment, the Myeloma XI study included a maintenance arm consisting of lenalidomide + vorinostat in which vorinostat was dosed at 300 mg/day on days 1–7 and 15–21 of the 28-day cycle [10]. However, higher rates of dose modifications and early discontinuation of maintenance therapy in the combination arm led to the protocol amendment closing the combination arm and patients switching to lenalidomide alone. Among the 614 patients (395 transplant-eligible and 219 transplant-ineligible) randomized in a 1:1 manner to either lenalidomide or lenalidomide + vorinostat, the median PFS was 40.2 months and 33.1 months, respectively, with a median follow-up of 41.8 months [28]. When accounting for dose modification, the PFS HR was 0.99 (95% CI, 0.98–1.00) while the OS HR was 0.90 (95% CI, 0.83–0.98) [28].

### 3.5. Lenalidomide + Anti-CD38 Monoclonal Antibody Therapy

Multiple studies are investigating the impact of adding daratumumab or isatuximab to lenalidomide maintenance post-ASCT. Thus far, preliminary results have been reported from the maintenance portion of the GRIFFIN study. This randomized phase II study compared RVd induction/ASCT/RVd consolidation/lenalidomide maintenance to the experimental arm in which daratumumab was added to induction, consolidation, and maintenance. Initially, daratumumab was administered on an every 8-week schedule during maintenance, but a protocol amendment changed this to monthly dosing [29]. The duration of maintenance therapy was up to 2 years on study, after which it was recommended that patients continue single-agent lenalidomide as per standard of care. After 12 months of maintenance, a deepening of responses was observed in both treatment groups, however the daratumumab-containing arm had superior rates of stringent CR (63.6% vs. 47.4%; *p* = 0.0253) and MRD negativity (sensitivity 1 *×* 10^−5^; 62.5% vs. 27.2%; *p* < 0.0001) compared to the control arm (lenalidomide maintenance) [30]. 

There are several ongoing phase III maintenance studies as well as induction/ASCT/consolidation/maintenance studies that will provide greater insight into the long-term benefit of incorporating anti-CD38 mAb therapy. The ongoing SWOG S1803 study randomizes patients who have undergone ASCT to either lenalidomide alone or in combination with daratumumab. The study has a primary endpoint of OS and is powered to compare a median OS of 10 years in the lenalidomide arm compared to 15.7 years in the combination arm. This study will require the enrollment of 1100 patients over a six-year period. There is a response-adapted treatment decision following completion of two years of maintenance therapy: those patients who are MRD-positive will continue their assigned maintenance treatment. However, patients who are MRD-negative at that time point will be randomized to either stopping maintenance therapy or continuing it. Daratumumab (subcutaneous formulation) is administered weekly for two cycles, every other week for four cycles, and then once every 28 days thereafter. 

The phase III MMY3021/AURIGA study is assessing whether the percentage of patients converting to MRD negativity (10^−5^ sensitivity) after 12 months of maintenance is improved with the addition of daratumumab to lenalidomide. This trial will enroll 214 patients, who are MRD-positive after upfront ASCT. A similar schedule of daratumumab is used as in the SWOG S1803 study. In both arms, maintenance treatment will be continued for up to 36 months. The phase III PERSEUS study was designed to assess the addition of daratumumab to RVd induction/consolidation. Patients randomized to RVD will receive lenalidomide maintenance until progression or unacceptable toxicity. Patients randomized to Dara-RVd will receive daratumumab/lenalidomide maintenance until progression unless they achieve sustained MRD negativity for 12 months and after a minimum of 24 months of maintenance therapy. In that case, the daratumumab is discontinued while lenalidomide is continued until progression. However, if MRD negativity is lost or there is relapse from CR, then daratumumab is resumed and the doublet is continued until disease progression or unacceptable toxicity. Thus, the slightly different study designs will make it difficult to perform cross-trial comparisons, but will provide insight into the potential benefit of combination therapy based on MRD status.

### 3.6. Lenalidomide + Elotuzumab

Thomas et al. reported the preliminary results of a phase II study evaluating lenalidomide in combination with the anti-SLAMF7 monoclonal antibody elotuzumab as maintenance therapy post-ASCT [31]. Elotuzumab was initially administered 10 mg/kg weekly for two cycles, every other week for four cycles, and then 20 mg/kg once-monthly. Subsequently, this was changed to 20 mg/kg once-monthly dosing starting on cycle 3. Amongst the first 55 patients enrolled, 44% achieved a deepening of response while on maintenance therapy [31]. Subsequent analyses with additional patients and longer-term follow-up found a three-year PFS of 81%, which was inferior in the high-risk cytogenetic cohort [32]. A more recent presentation included 100 patients with an estimated four-year PFS of 75% with 27% of patients converting to CR while on therapy [33]. The ongoing GMMG HD6 trial (NCT02495922) is evaluating the addition of elotuzumab to the RVd induction and/or consolidation setting. Patients will receive either single-agent lenalidomide or lenalidomide in combination with elotuzumab maintenance for up to two years. The results of the induction period are such that rates of VGPR or better following four cycles of induction were not improved by the addition of elotuzumab [34]. Additional follow-up is needed for the analysis of the maintenance component.

## 4. Second Primary Malignancies

As the CALGB 100104 and IFM 2005 studies progressed, a signal began to emerge for an increased occurrence of SPMs in patients receiving post-ASCT lenalidomide maintenance. The IFM study chose to amend the protocol and discontinue maintenance, while the CALGB study continued protocol therapy. The McCarthy meta-analysis reported frequencies of 5.3% and 0.8% for hematological SPMs occurring before MM disease progression in the lenalidomide and placebo/observation arms, respectively (CALGB/IFM) [11]. While the time to diagnosis of an invasive SPM (occurring prior to MM disease progression or start of second-line therapy) was shorter in the placebo/observation group (HR, 2.67; 95% CI, 1.54–4.62; *p* < 0.001), the time to MM progression/second-line therapy was longer with lenalidomide (HR, 0.51; 95% CI, 0.45–0.59; *p* < 0.001) and the overall risk of MM disease progression was higher than the risk of developing an invasive SPM [11]. Furthermore, the time to death secondary to MM was longer in the lenalidomide group (HR, 0.66; 95% CI, 0.53–0.81; *p* < 0.001), but there were no differences between the two groups with respect to time to death as a result from an SPM or an adverse event [11]. The Myeloma XI trial group reported a three-year cumulative incidence of 5.3% in the lenalidomide group vs. 3.1% in the observation group (HR, 1.85; 95% CI, 1.18–2.90), but this was not further broken down into the TE vs. TI cohorts [10]. Whether addition of other agents to the lenalidomide backbone will significantly alter the SPM risk remains to be determined. With relatively short follow-up from the GRIFFIN study, no hematological SPMs have been reported in either arm [29,30].

It is recognized that having a diagnosis of a plasma cell dyscrasias [35,36,37], as well as undergoing a high-dose melphalan ASCT, is associated with an increased risk of hematological SPMs [38]. It has been hypothesized that lenalidomide exposure in the context of alkylating therapy may lead to an increased risk of SPMs. A meta-analysis involving 3254 newly diagnosed patients from seven phase 3 studies showed a five-year SPM incidence of 6.9% (lenalidomide) vs. 4.8% (no lenalidomide) (*p* = 0.037), with the increased risk being a consequence of hematological malignancies and not solid tumors [39]. In this meta-analysis, there was an increased risk of hematological SPMs associated with lenalidomide and low-dose oral melphalan but not with higher-dose IV melphalan. A report from the Connect MM^®^ registry evaluated the incidence of SPMs in four exposure comparison groups: exposed to lenalidomide or not, received ASCT with or without lenalidomide maintenance, exposed to melphalan or not, and exposed to oral melphalan with or without lenalidomide (within 160 days) [40]. No significant differences in the three-year cumulative probability of either hematological or solid tumor SPMs were found between any of the four exposure comparison groups [40]. An analysis of the CIBMTR database involving over 4100 subjects found that post-ASCT use of either thalidomide (15% of subjects) or lenalidomide (11% of subjects) was not significantly associated with the overall risk of developing an SPM [41]. The overall risk of developing an invasive SPM after ASCT was similar to that of the general population (age-/gender-/race-matched) with the exception of increased risk of myeloid malignancies and melanoma [41]. 

Thus, the extent to which lenalidomide maintenance post-ASCT increases the risk of SPMs is not yet fully understood and neither are the potential underlying mechanisms. In a study that involved targeted sequencing of the stem cell product of over 600 patients with MM undergoing ASCT, 21.6% were identified as having clonal hematopoiesis of indeterminate potential (CHIP). Twenty-one patients who had received thalidomide or lenalidomide maintenance developed secondary MDS/AML. An association with IMiD maintenance and subsequent therapy-related myeloid neoplasm (TMN) was noted (*p* = 0.047), but the presence of CHIP prior to ASCT was not associated with increased risk of TMN (*p* = 0.4) regardless of whether IMiD maintenance was administered [42]. Thus, the role for routine screening of CHIP prior to ASCT or initiation of maintenance is not yet defined. Finally, there have also been cases of acute lymphoblastic leukemia reported in association with lenalidomide maintenance [6,8], and it remains to be determined whether the risk factors for that SPM are distinct from those of the myeloid SPMs. 

## 5. Optimal Duration of Maintenance Therapy

The optimal duration of maintenance therapy remains to be determined, and is likely not a one-size-fits all answer. In the case of the phase III lenalidomide maintenance studies, the intent was to treat until progression (or adverse event). Concerns regarding the number of SPMs reported on the lenalidomide arm led the IFM 2005 trial to discontinue study maintenance for all patients. Patients had received 1–3 years of treatment at that time (median of 2 years). In the CALGB 100104 study, the median duration of treatment was 31.0 months (95% CI, 24.8–35.8) although the median PFS was 57.3 months (95% CI, 44.2–73.3), highlighting that the majority of patients discontinued protocol maintenance therapy prior to disease progression [8]. In the real-world setting, greater flexibility with respect to dose reductions and schedule may enable patients to stay on treatment longer. As an example, the Emory group reported that the median duration of maintenance therapy (primarily lenalidomide) post-upfront ASCT was 57 months (range, 49.9–64.1 months) [22]. A recent updated analysis of the BMT CTN 0702 study provides some insight into the importance of duration of lenalidomide maintenance. This study was originally designed such that all three groups (single ASCT, tandem ASCT, or single ASCT followed by RVd consolidation) were to receive lenalidomide maintenance for 3 years. However, in 2014, the protocol was amended to allow patients to continue on lenalidomide maintenance until progression. A landmark analysis of patients who chose to stop lenalidomide (n = 207) vs. those who continued beyond the ~three-year time point (n = 215) demonstrated superior PFS for those who remained on lenalidomide (e.g., five-year PFS 67.2% vs. 86.5%) [43]. The phase III GMMG-MM5 trial, which compared PAD to bortezomib/cyclophosphamide/dexamethasone induction prior to ASCT, also included two cycles of full-dose lenalidomide consolidation followed by either two years of lenalidomide maintenance (Len-2Y) or cessation of lenalidomide in patients achieving a CR before the start of maintenance or during maintenance (Len-CR) [44]. The PFS did not different between the two maintenance strategies (HR, 1.15; 95% CI, 0.93–1.44; *p* = 0.2), but the OS was superior in the Len-2Y group (HR, 1.42; 95% CI, 1.04–1.93; *p* = 0.03). In particular, patients who achieved a CR after lenalidomide consolidation and thus never initiated maintenance had inferior PFS (HR, 1.84; 95% CI, 1.08–3.13; *p* = 0.02) [44].

The phase III studies involving ixazomib [17] or daratumumab [20] utilized fixed-duration therapy, but whether two years is the optimal duration has yet to be demonstrated. Several studies have demonstrated that maintenance therapy improves outcomes even in patients who achieve MRD negativity at day 100 post-ASCT [17,45]. There is significant interest in determining whether MRD status can guide treatment duration/escalation/de-escalation in the post-ASCT maintenance setting. Data from ongoing trials will provide insights into the role of MRD in guiding treatment decisions in the maintenance space. The existing body of data support the recommendation that the intent of treatment should be to continue lenalidomide maintenance until disease progression. 

## 6. Maintenance Trial Design

Survival outcomes for newly diagnosed TE patients continue to improve. The median OS from the start of ASCT in the lenalidomide arm in CALGB 100104 (a study conducted prior to addition of triplet induction regimens such as RVd into the treatment armamentarium) was 9.5 years [8]. In that study, the start of ASCT was typically 6–12 months after initiation of induction therapy. In the report of the Emory experience, the median OS of the entire cohort of newly diagnosed patients treated with RVd induction (of whom approximately 75% received upfront ASCT) was 10.5 years [22]. The GIMEMA-MMY-3006 study randomized patients to either VTD (bortezomib, thalidomide, and dexamethasone) or TD (thalidomide and dexamethasone) as induction/consolidation around tandem ASCT. After a median of 10 years of follow-up, the median OS was 9.2 years for the TD arm, but had not yet been reached for the VTD arm [46]. 

Thus, it is evident that the current median OS benchmark is at least 10 years. With all of the recent approvals, including chimeric antigen receptor (CAR) T-cell therapy, it is quite likely that the median OS will continue to improve. With each incremental improvement in OS, it becomes more challenging to assess the clinical benefits of experimental treatments due the longer study durations, larger sample sizes, and increases in study-related costs. An example of this is the ongoing SWOG S1803 study mentioned above, which plans to enroll 1100 patients to assess whether the addition of daratumumab to lenalidomide will increase median OS from 10 years to approximately 16 years. Other issues encountered with long clinical trials that may impact the interpretation of study results include the types of salvage treatments utilized early in the follow-up period varying considerably from that utilized later in the follow-up period, patients assigned to the standard treatment arm possibly receiving the experimental treatment later in their disease course, and the percentage of patient who withdraw possibly creeping higher as the study goes on. The possibility exists that by the time data are mature, there may have been a number of clinical findings eroding the impact of the results of the trial on clinical decision-making.

One area of research is developing surrogate endpoints for OS in MM. Appropriateness of a surrogate endpoint may depend on the line of treatment or the nature of the treatment. There have been no meta-analyses with patient-level data assessing the validity of PFS as a surrogate endpoint for OS in MM. PFS as a clinical endpoint has its own set of challenges. Estimates of PFS are impacted by surveillance schedules and evaluation methods utilized in the trial as well as the analytic procedures, such as censoring schemes and the handling of competing events such as second primary cancers and death without disease progression. A key area of interest in the field is to have a validated endpoint of clinical benefit that can be read-out early in the course of treatment. One endpoint that is being investigated as a potential surrogate endpoint is MRD negativity. There have been multiple reports that achievement of MRD negativity is associated with prolonged PFS/OS outcomes [47,48,49,50]. However, a number of issues must be resolved for MRD to gain designation as a surrogate endpoint from a regulatory perspective, including the optimal MRD assessment methodology, sensitivity of assay, timing of assessments, duration of sustained MRD negativity, and the incorporation of disease assessment outside of the bone marrow (i.e., with advanced imaging) [51]. There is an ongoing collaboration between academia and industry representatives (International Independent Team for Endpoint Approval of Myeloma MRD (i^2^TEAMM)) that is attempting to establish an MRD-based endpoint as a surrogate for PFS [52].

## 7. Moving beyond ASCT

The current body of literature supports the continued role for upfront ASCT as this has been associated with superior PFS compared to chemotherapy alone. A recent meta-analysis of four randomized phase III studies revealed a combined HR of 0.55 for PFS (95% CI, 0.41–0.74; *p* < 0.001) and 0.76 for OS (95% CI, 0.42–1.36; *p* = 0.20) in favor of the upfront ASCT group [53]. In addition, the most recent update from the phase II FORTE study reported superior PFS (HR, 0.64; *p* = 0.023) for the KRD/ASCT arm compared to the non-transplant arm that received KRD induction [25]. However as induction regimens continue to evolve (e.g., quadruplet combinations such as Dara-RVD [29] or Dara-KRd [54]), as well as the advent of new cellular therapies (such as CAR T-cells [55]) and bispecific agents [56,57], the question remains whether routine use of upfront ASCT will diminish over time. In that context, the question then becomes what the role for maintenance therapy will be without ASCT. It is interesting to note that several preclinical studies have demonstrated that IMiDs as well as cereblon E3 ligase modulation drugs (CELMoDs) enhance CAR T-cell activity [58,59,60]. As a late line of therapy, BCMA CAR T-cell therapy does not appear to be curative, and thus incorporation of an IMiD or CELMoD in the maintenance setting post-CAR T-cell may improve outcomes. Whether this approach would also be of benefit in the newly diagnosed setting also merits investigation.

## 8. Conclusions

While post-ASCT maintenance therapy with lenalidomide continued until disease progression is the current standard of care based on the available clinical trial data, ongoing studies will likely lead to practice changes. The most likely next step will be to incorporate anti-CD38 monoclonal antibody therapy into the maintenance setting, but it remains to be determined whether all patients will benefit from multi-agent approaches. Presently there are not sufficient data to guide the selection of lenalidomide/anti-CD38 monoclonal antibody therapy for specific patient populations (e.g., high-risk cytogenetics or MRD-positivity post-ASCT) outside of the context of a clinical trial. The study design of some of the ongoing trials may provide insight as to whether response-adapted approaches (e.g., using MRD status) can guide treatment duration. Ultimately the goal would be to tailor maintenance therapy (both from an agent selection perspective and from a duration perspective) based on an individual patient’s disease characteristics, including features such as response (such as MRD status), cytogenetic/genomic abnormalities, and even immune microenvironment characteristics. Such a personalized medicine approach to maintenance therapy would therefore have the potential to maximize disease response and survival outcomes while minimizing treatment burden.

## Figures and Tables

**Table 1:** Summary of randomized phase III trials evaluating lenalidomide maintenance after ASCT.

Study	N	Induction Therapy	Dosing Schedule	Intended Duration of Maintenance	Reported Duration of Lenalidomide Maintenance	TTP or PFS (Maintenance vs. No)	OS (Maintenance vs. No)	SPMs
CALGB 100104 [7,8]	460	≤2 regimens; 94% received a regimen containing Thal, Len, and/or Bor	10 mg continuous, increase up to 15 mg	Until progression	31 months (median)	Median TTP *: 57 vs. 29 months (HR, 0.57; *p* < 0.0001)	Median OS *: 114 vs. 84 months (*p* = 0.0004)	Len: 8% hematologic, 6% solid tumor, 5% noninvasive Pbo: 1% hematologic, 4% solid tumor, 3% noninvasive
IFM 2005-02 [6,11]	614	46% received vincristine, doxorubicin, Dex and 46% received Bor and Dex	All patients received 2 cycles of consolidation (25 mg/d, 21 out of 28 days)	Stopped due to concerns regarding second primary malignancies at a median time of 2 years (range 1–3 years)	25 months (mean)	Median PFS: 41 vs. 23 months (HR, 0.50; *p* < 0.001)	Median follow-up 45 months: 74 vs. 76% (*p* = 0.7)	Len: 4% hematologic, 3% solid tumor, 2% nonmelanoma skin cancer Pbo: 2% hematologic, 1% solid tumor, 1% nonmelanoma skin cancers
		21% received tandem transplant	Maintenance: 10 mg continuous, increase up to 15 mg			4-year PFS: 43 vs. 22% (*p* < 0.001)	4-year OS: 73% vs. 75% (*p* = 0.7)	
RV-MM-209 [9,11]	402	4 cycles Len/Dex followed by either transplant or MPR	10 mg (21 out of 28 days)	Until progression	35 months (mean) (TE population)	Median PFS **: 42 vs. 22 months (HR, 0.47; *p* < 0.001)	3-year OS **: 88% vs. 79% (*p* = 0.14)	4.3% (Len) vs. 4.3% (Obs)
Myeloma XI [10]	1247 ***	CTD vs. RCD followed by CVD if suboptimal response	10 mg (21 out of 28 days)	Until progression	NR for TE population	Median PFS: 57 vs. 30 months (HR, 0.48; *p* < 0.0001)	3-year OS: 88 vs. 80% (HR, 0.69; *p* = 0.014)	3-year cumulative incidence: 5.3% (Len) vs. 3.1% (Obs) ****

* Placebo group includes 86 patients who chose to cross over to receive lenalidomide at time of study unblinding; ** Combining ASCT and chemotherapy groups; *** Transplant-eligible only (total number in the study was 1970); **** For the entire study population; data not reported for the transplant-eligible population; Abbreviations: Bor (bortezomib), CTD (cyclophosphamide, thalidomide, dexamethasone), CVD (cyclophosphamide, bortezomib, dexamethasone), Dex (dexamethasone), HR (hazard ratio), Len (lenalidomide), MEL200 (melphalan 200 mg/m^2^), MPR (melphalan, prednisone, lenalidomide), NR (not reported), Obs (observation), OS (overall survival), Pbo (placebo), PFS (progression-free survival), RCD (lenalidomide, cyclophosphamide, dexamethasone), TE (transplant eligible), Thal (thalidomide), and TTP (time to progression).

## Data Availability

Not applicable.
